# Recent progress in magnetic applications for micro- and nanorobots

**DOI:** 10.3762/bjnano.12.58

**Published:** 2021-07-19

**Authors:** Ke Xu, Shuang Xu, Fanan Wei

**Affiliations:** 1 School of Information & Control Engineering, Shenyang Jianzhu University, Shenyang, China; 2 School of Mechanical Engineering and Automation, Fuzhou University, Fuzhou, China

**Keywords:** magnetic drives, magnetic nanoparticles, magnetoelectric devices, micro- and nanorobots

## Abstract

In recent years, magnetic micro- and nanorobots have been developed and extensively used in many fields. Actuated by magnetic fields, micro- and nanorobots can achieve controllable motion, targeted transportation of cargo, and energy transmission. The proper use of magnetic fields is essential for the further research and development of micro- and nanorobotics. In this article, recent progress in magnetic applications in the field of micro- and nanorobots is reviewed. First, the achievements of manufacturing micro- and nanorobots by incorporating different magnetic nanoparticles, such as diamagnetic, paramagnetic, and ferromagnetic materials, are discussed in detail, highlighting the importance of a rational use of magnetic materials. Then the innovative breakthroughs of using different magnetoelectric devices and magnetic drive structures to improve the micro- and nanorobots are reviewed. Finally, based on the biofriendliness and the precise and stable performance of magnetic micro- and nanorobots in microbial environments, some future challenges are outlined, and the prospects of magnetic applications for micro- and nanorobots are presented.

## Introduction

Micro- and nanorobots (MNRs) present challenges and prospects in the field of nanotechnology. MNRs have been a major direction of technological development and will be widely used in many fields such as biomedicine, electronic technology and sensing, and environmental remediation [1–4]. Therefore, research on MNRs has become the pursuit of many researchers [5]. In recent years, a variety of driving methods for MNRs have been proposed, such as light [6–9], acoustics [10–12], magnetic fields [13–20], self-electrophoresis and self-thermophoresis, bubbles, and many other forms [21]. As a biofriendly approach, the penetration of biological tissues without damage and free movement in the microbial environment can be achieved with magnetic fields, which has become a highlight of research in the field of MNRs. A magnetic microrobot (MMR) [14] is a micro-/nanoscale device with embedded magnetic materials. The locomotion of MMRs is actuated and controlled through the principles of magnetism regarding energy transfer and the magnetoelectric effect.

Magnetic materials have been widely used in the field of MNRs to control and drive the movement of robots, target the transportation of cargo [22–23], and transmit energy. Compared with other concepts, magnetic MNRs that combine diamagnetic, paramagnetic, and ferromagnetic materials [24] could have a greater driving force and exhibit characteristics such as biocompatibility [25]. Hence, magnetic nanoparticles (MNPs) are widely used in medical MNRs [26]. For example, based on the combination of copolymer brushes and superparamagnetic nanoparticles, a biomimetic nanoreactor was proposed. It contained a magnetic field-responsive catalytic system, namely magnetic field-responsive binary deoxyribozyme (MaBiDZ) [27]. Also, it showed stability, specificity, and semi-permeability, together with cell compatibility [25], which could be well applied to MNRs and enhance the driving force of MNRs under certain conditions. Energy transmission and control of the MNRs could be realized by wireless power transfer (WPT) systems [16]. The advantages of electromagnetic drives as a power source are no heat, no electrical contacts, good controllability, and high durability. Through the coupling of magnetic and electric fields, motion control of the MNR could be realized. When studying the combination of magnetic and electrical systems of MNRs, it is necessary to advance from multiple angles at the same time and improve energy transmission efficiency while also improving other aspects of performance as much as possible. For magnetic MNRs, a specific magnetic drive structure is required. For example, a MNR with conical spiral structures could be efficiently processed by modulated structured light. The MNR with this structure had a stronger surge performance and the ability to load cargo. Magnetically responsive materials were added to the surface of a MNR with a structure of a hollow helical conically shaped tube. A custom-built three-dimensional Helmholtz coil control system was used to form a rotating magnetic field in three dimensions. A change in the direction of the magnetic field exerted a magnetic moment to steer the magnetic structure. Compared with the traditional straight helical structure for MNRs [28], the conical helical structure had a higher motion speed and could effectively suppress lateral drifting motion. In addition, MNRs with a hollow tubular structure [29], which could facilitate drug delivery and realize effective treatment of cancer by loading and releasing anticancer drugs, were proposed and fabricated. At the same time, a change of the magnetic field gradient and direction can be used to accurately guide magnetic microstructures. However, these structures often have some shortcomings and limitations. Different magnetic drive structures show different characteristics. Research is needed to continuously improve and perfect micro- and nanorobotic systems. A combination of magnetic drive and micromixer can also be applied to MNRs. For example, there have been a variety of electromagnetic micromixers, including external permanent magnets, electromagnets, microstirrers, and integrated electrodes. The following article describes the specific content in detail.

Researchers have approached to the topic of MNRs from different perspectives. The article analyzes magnetic materials, the combination of magnetism and electricity, and the structures of magnetic drives. Exploration and research of magnetic fields will promote the applications of MNRs in nanotechnology. This article focuses on recent progress in magnetic applications of MNRs. This article is organized as follows: After the description of magnetic materials adopted in MNRs, magnetoelectric concepts used in MNRs and structures of magnetic drives used in MNRs will be discussed. Finally, conclusions and an outlook will be provided.

## Review

### Magnetic materials used in MNRs

Different magnetic materials exhibit different magnetic properties to external magnetic fields. When analyzing MNPs [30] in medical nanorobots, Martel [26] mentioned that according to the alignment and response of magnetic dipoles, magnetic materials can be divided into diamagnetic, paramagnetic [31], ferromagnetic, ferrimagnetic, and antiferromagnetic. Diamagnetism of the material can be attributed to the orbital angular momentum, which is a phenomenon in which nanoparticles gain magnetization against an applied external magnetic field. Paramagnetism is caused by spin angular momentum (i.e., spin magnetic moment).

Under the action of an external magnetic field, the initially disordered magnetic moments will be reoriented, thereby exhibiting paramagnetism, while other forms of magnetism (ferromagnetism, ferrimagnetism, or antiferromagnetism) are produced by the interaction of magnetic dipoles. MNRs with the addition of magnetic materials can be remotely actuated in water, blood, and even cell tissue fluid. Magnetic MNRs have been employed to conduct research in the field of medicine. Zhang et al. [32] used polymer-conjugated magnetic nanoparticles as a MNR to effectively carry nucleic acids in vitro. Their team developed a polyethylenimine-conjugated magnetic nanomaterial. While exhibiting long-term stability, this material can realize redox-activated and magnet-assisted gene transfection. Magnetic MNRs containing nucleic acids were delivered to target cells by magnetic fields. This method could change gene function or protein expression, which is of great significance for future research on gene transfer and gene therapy. Table 1 summarizes the relevant properties of magnetic materials for MNRs. Currently, mainly paramagnetic [33] and diamagnetic [34] nanoparticles are used. Next, we will focus on these two classes of materials.

**Table 1:** Magnetic materials for MNRs.

Magnetism	Principle of magnetism	Macroscale strength of magnetism	Magnetic susceptibility	Permeability	Advantages	Ref.

diamagnetic	orbital angular momentum	weak magnetism	χ < 0, around −10^−7^ to −10^−6^, does not change with temperature	μ_Υ_ < 1 (μ_Υ_ = 0 represents a superconductor)	compared with permanent magnets, there no friction and 3-D control	[34,47–48]
paramagnetic	spin angular momentum	weak magnetism	χ > 0, around 10^−6^ to 10^−5^, increases with decreasing temperature	μ_Υ_ > 1	compared with ferromagnetic particles, there is no magnetization and coercivity	[27,35,40–42]
ferromagnetic	exchange interaction of magnetic dipoles	strong magnetism	χ > 0, around 10^−1^ to 10^5^, ferromagnetic at temperatures lower than the Curie temperature, paramagnetic at temperatures higher than the Curie temperature	μ_Υ_ ≫ 1	low power consumption, room temperature adaptability, self-stability, easy to miniaturize	[26,49–51]
ferrimagnetic	Exchange interaction of magnetic dipoles	strong magnetism	χ > 0, around 10^−1^ to 10^4^, ferrimagnetic at temperatures lower than the Curie temperature, paramagnetic at temperatures higher than the Curie temperature	—	low toxicity	[26]
antiferromagnetic	exchange interaction of magnetic dipoles	strong magnetism	χ > 0, around 10^−5^ to 10^−3^, antiferromagnetic at temperatures lower than the Néel temperature, paramagnetic at temperatures higher than the Néel temperature	—	—	[26,53]

#### Paramagnetic nanoparticles

Paramagnetic nanoparticles [35] can be used for drug delivery with MNRs. When exposed to external magnetic fields, the interaction between paramagnetic nanoparticles might lead to the formation of chain structures [36]. Although external magnetic fields can be used to effectively gather and transport paramagnetic nanoparticles, they tend to form aggregations. Therefore, assembly and disassembly processes of MNPs are adopted to fabricate MNRs [37]. A rotating magnetic field was often used to break particle chains into shorter fragments. As the magnetic field changes, the particle chain will be broken and re-formed. While disassembling the paramagnetic nanoparticle chains of MNRs, it is necessary to avoid the reassembly of the chains. Doherty et al. [38] pointed out that superparamagnetic nanofibers could prevent the uncontrolled agglomeration of particles because the residual magnetization of this material is almost zero. They applied this technology to sensing and environmental remediation and prepared framework composites by growing MOF-5 crystals on ferrite nanofibers to remove impurity particles with the help of the embedded magnetic nanofibers. The realized the extraction of carcinogenic molecules, such as polycyclic aromatic hydrocarbons (PAHs), with magnetic framework composites (FCs). Nayak [39] used metal-organic frameworks (MOFs) to adsorb heavy metals in water for water purification. MOFs have a very high specific surface area and modular structure, showing great advantages in the sustainable supply of clean drinking water.

Later, Yu et al. [40] reported a method to disassemble paramagnetic nanoparticle chains. They used a predefined dynamic magnetic field that could controllably spread and fragment the particle chains. This is an effective strategy that shows that the assembly and disassembly process is reversible. Swarms of MNR paramagnetic nanoparticles moved together in the form of clusters in the microvasculature with high velocity, actuating forces, and accessing rates. In addition, the swarms can be broken up when necessary to enter the microvasculature and avoid thrombosis. Length and distribution of the fragmentation were controlled, and the process was reversible. At the same time, in order to reduce the chance of reorganization of the fragments a distance between disassembled parts could be established. Through magnetically induced repulsive forces the nanoparticle chains were forced to diffuse in different directions. This new method is of great significance for understanding magnetic MNR swarms, and further applications may be found.

**Figure 1:**
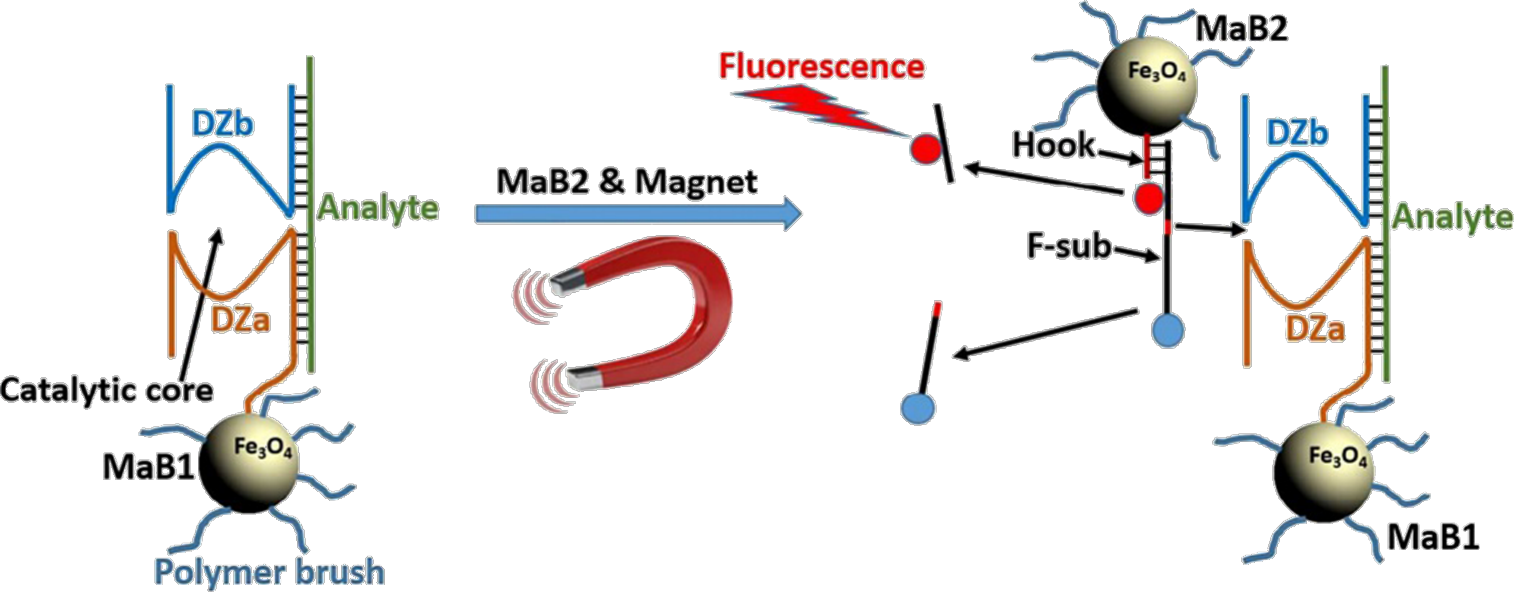
Working principle of MaBiDZ. Magnetic bead (MaB1)-bound DZa forms a catalytic core with DZb in the presence of analyte. The magnetic bead (MaB) architecture is composed of a 15 nm iron oxide (Fe_3_O_4_) superparamagnetic core encased in a silica shell. The DNA strands are conjugated to the polymeric brush using a flexible linker. The brush permits passage of the nanoparticles through cell membranes, and prevents nanoparticle aggregation in the absence of a magnetic field. This allows the gated compartments to form only in the presence of a magnetic field (ca. 0.4 T), creating a stimuli-responsive nanoreactor. The activated nanoreactor produces a signal only when (i) a second species of magnetic beads, MaB2 carrying F-sub, is present and (ii) a magnetic field that aggregates MaB1 and MaB2 is applied. Adapted with permission of the Royal Society of Chemistry, from [27]; (“Nanoreactors Based on DNAzyme-Functionalized Magnetic Nanoparticles Activated by Magnetic Field” by S. F. Bakshi et al., Nanoscale, vol. 10, issue 3, Copyright © 2018); permission conveyed through Copyright Clearance Center, Inc. This content is not subject to CC BY 4.0.

Bakshi et al. [27] reported a magnetic field-responsive catalytic system based on superparamagnetic nanoparticles [41], namely magnetic field-responsive binary deoxyribozyme (MaBiDZ, Figure 1), which could sense intracellular target mRNA and could be applied to MNRs. MaBiDZ is mainly composed of three parts, that is, the DZb strand, MaB1, and MaB2. MaB1 is conjugated with DZa, and MaB2 is conjugated with a DNA hook strand complementary to F-sub. An external magnetic field will make MAb1 and MAb2 aggregate, so that the activated BiDz sensor can approach F-sub, and then F-sub cutting and fluorescence signal amplification are carried out in the presence of analytes. Bakshi et al. proposed a cellular nanoreactor. It had two kinds of magnetic particles, each of which is functionalized by two components of the binary deoxyribozyme system. The nanoreactor was assembled in the presence of a target mRNA analyte and a magnetic field to form a biocompartment enclosed by the polymeric brush. Due to the polymer brush, MaBiDz exhibited excellent properties such as stability, specificity, and semi-permeability. It provides a suitable microenvironment to promote intracellular biocatalysis. MaBiDz dynamics were significantly enhanced compared with free BiDz. MaBiDz also has cytocompatibility, is easy to integrate with cells, and exhibits intracellular stability. These features make it possible to quickly detect breast adenocarcinoma biomarkers, with which cancer cells and non-cancerous cells can be distinguished. Furthermore, a cell sensor that uses MaBiDz for rapid detection and imaging of target mRNA biomarkers of metastatic breast cancer has been realized. Its function shows that it is likely to be used as a biomimetic organelle MNR in the field of biomedicine.

Ceylan et al. [42] also used superparamagnetic nanoparticles to explore 3D-printed biodegradable [17,24] microrobots. These robots could be used for theranostic cargo delivery and release. Embedding superparamagnetic iron oxide nanoparticles [43] in the form of nanocomposites into the microrobot will impart magnetizability. Magnetic field-based transport enables the accelerated delivery of a biomaterial to a target site by overcoming Brownian diffusion [44]. Since cobalt and nickel are quite toxic and iron oxide nanoparticles are considered to be biofriendly [45], embedding iron oxide nanoparticles [46] has advantages over magnetic surface coatings, such as cobalt or nickel.

#### Diamagnetic nanoparticles

Applying an external magnetic force to manipulate the MNRs has become a frontier field of research. Uvet et al. [47] proposed a new microrobot manipulation technology based on diamagnetic levitation nanomaterials. Without using strong electromagnets or bulky permanent magnets, it can make the microrobot move in three dimensions in a liquid environment through diamagnetic levitation. The main purpose of this method is to eliminate friction between the substrate surface and the microrobot. The position accuracy of this technology is extremely high and can reach the nanometer level. A permanent magnet was embedded in the microrobot, which will make the microrobot levitate under the action of a diamagnetic material. In this process, this was realized without an active control mechanism. In the method of Uvet et al., the levitation height of the microrobot was controlled by an external ring-shaped permanent magnet, and pyrolytic graphite (PG) was used to provide the balance force. The microrobot was composed of SU8 and permanent magnets. The direction of the 3D motion of the microrobot could be changed by the position of the lifter magnet. The advantage of the system is that it does not require current control and can use single carrier magnets and lifter magnets to control three-dimensional motion in a liquid environment, eliminating unwanted physical effects that might occur with other methods, such as heat and noise. Using this method, Uvet and others provided an innovative micro-UFO, which was suspended by diamagnetic levitation. After the micro-UFO reached a certain height, it could successfully track the maximum range of motion of the linear stage of 500 nm through the lifter magnet, a design suitable for biomedical applications.

Magnetic MNRs are a major topic in current research. Researchers continue to explore and apply properties of diamagnetic nanoparticles. Cho et al. [34] carried out research on diamagnetic nanoparticles. They analyzed a low-level radiation shielding sheet with diamagnetic nanoparticles and wanted to develop a medical radiation fiber shielding, which is harmless to the human body. The uniform dispersion of magnetic nanoparticles into a polymer resin can not only reduce the weight of the material, but also avoid harm to the human body or the environment caused by other materials such as lead. In addition, Gopal et al. [48] pointed out that boron exhibits diamagnetic properties in B-doped anatase TiO_2_ nanoparticles and showed photocatalytic activity in the visible-light range. Magnetic MNRs were applied to the chemical field, and preparation and characterization of B–TiO_2_ photocatalytic particles were carried out by using these diamagnetic nanoparticles. The research on magnetic nanoparticles will expand the application range of MNRs and promote the common development of different fields.

#### Other nanoparticles

Ferromagnetic, antiferromagnetic, and other nanomaterials are also used in MNRs. Martel et al. [49] have demonstrated that magnetotactic bacteria embedded in ferromagnetic particles can be used as medical MNRs to control MRI-trackable propulsion and can be used in human microvascular environments. David Folio [50] also proposed a navigation method for two-dimensional robust magnetic resonance of microrobots using ferromagnetic nanoparticles. Laurent Arcese et al. [51] discussed the control design of an intravascular magnetically guided microrobotic system for performing minimally invasive medical procedures. The microrobot they designed contained aggregates of ferromagnetic nanoparticles to carry loads driven by gradient magnetic coils. With the help of ferromagnetic nanoparticles, a preliminary study on the navigation method of microrobots in a fluidic environment was carried out, and an innovative method to complete medical tasks with the optimal trajectory of controllable movement of MNRs in cardiovascular system was proposed.

Some multiferroic nanomaterials exhibit ferromagnetism and antiferromagnetism by adjusting electricity and magnetism. A variety of multifunctional devices can be prepared with different magnetisms with applications in, for example, energy-saving logic, sensors, environmental remediation, and data storage [52]. Chen et al. [53] studied compensated magnetic heterostructures containing ferrimagnetic CoGd alloys and antiferromagnetic IrMn layers. The terahertz emission from the ferrimagnetic nanoparticles showed good temperature stability. The proposed new terahertz technology can be better applied to many fields such as chemical composition analysis and integrated circuit analysis. Hence, it can be seen that magnetic MNRs are widely used.

From controlling individual microparticles to more complex structures, deeper exploration and research on MNRs are gradually realized. In 2012, Falcaro et al. [54] proposed the use of external magnetic fields to locate single metal-organic framework nanoparticles, leading to the potential application of magnetic nanoparticles as active materials for microdevices (e.g., microchannels and microfluidic circuits). Recently, scientists began to explore more complex structures of magnetic MNRs, such as flagella-driven magnetic microswimmers and magnetic micro- and nanomotors. In the article of Zhou et al. [55], several kinds of MNRs driven by magnetic force and the development of a micromotor driven by magnetic force were discussed. Yang et al. [56] and Chen et al. [57] also analyzed and summarized magnetic MNRs. Next, MNRs with different magnetoelectric concepts and morphologies will be discussed.

### Magnetoelectric concepts applied to MNRs

Magnetic and electric fields are the main ways to remotely drive and control medical MNRs [58–59]. Compared with other unfettered transmission methods (such as light and chemical fuels), magnetic fields provide biocompatible energy and can safely catalyze MNRs that rely on non-biocompatible fuels for propulsion [58]. Catalytic converters that use biocompatibles fuel to move cannot move in biologically relevant ionic media [60]. Therefore, hybrid magnetoelectric drives [13] have become a main structure of MNRs. Table 2 shows several different forms of magnetoelectric concepts.

**Table 2:** Magnetoelectric concepts.

Magnetoelectric concept	Power generation	Functions	Object of action	Advantages	Ref.

electromagnetic actuation (EMA)	applying a current to a coil generates a magnetic field; applying a voltage to an electrode generates an electric field	MNRs can be manipulated to pick up and release particles	acts on electroactive hydrogels (easy to manufacture and quickly respond to small stimuli) and MNPs (support magnetic field drive)	could be actuated by electric fields and magnetic fields simultaneously; electroactive hydrogels are more accurate and programmable	[64]
wireless power transfer (WPT)	the resonance frequency is applied to achieve the maximum power transfer efficiency; the addition of magnetic materials will increase the magnetic coupling, and the bar-type magnetic material achieves a higher magnetic field gradient	generate propulsion, torque and transmit electrical energy; by adjusting the angle of the incident magnetic field and the magnetic material, the microrobot achieves a rotation motion	acting on bar-type coils and magnetic materials	can use LC resonance frequency, works independently of the operating frequency, and can be applied to any operating frequency range; the propulsion speed and transmission efficiency are very high	[71]
hybrid magnetoelectric (ME) nanowires	combination of a magnetostrictive core and a piezoelectric shell	wireless locomotion through a single external power source (magnetic field); precise steering toward a targeted location through magnetic fields; magnetoelectrically assisted drug release	precise control of the movement of the nanowire robot	flexibility in design and fabrication through core–shell configuration; biocompatibility and little side effects on healthy tissues during drug delivery	[76]
plug and play (PnP) electromagnetic coil system (MagDisk)	consists of five independent coils, can generate the required rotating magnetic field	actuation, control and observation of the fluorescent magnetic spore-based microrobot (FMSM), which can be easily integrated into a fluorescence microscope	actuation and control of the FMSM	PnP and low cost; the maximum output magnetic field strength is around 20 mT, which is enough to actuate the FMSM with a tumbling motion of more than 20 Hz	[77]

Electromagnetic driven microrobots could achieve certain motion under the influence of simultaneous electric and magnetic fields [61]. An electromagnetic actuation (EMA) [62–63] system could be used to locate microrobots containing materials with MNPs. Therefore, the combination of magnetism and electricity has been widely used in the design of various magnetic MNRs. Kim et al. [64] proposed a hydrogel microrobot based on the combination of an electroactive hydrogel and MNPs. Electroactive hydrogels are usually made of a single hydrogel [65]. They respond to electric fields and produce mechanical motion in an electrolyte [66]. Also, they respond more sensitively than other stimuli-responsive hydrogels [67]. The proposed microrobot had two arms, both of which were composed of anodic and cathodic electroactive hydrogels and MNPs. So they could be driven in a magnetic field and bent in an electric field to pick up and release cargo. Magnetic field gradients are applied to control the movement direction. To transport goods in an environment with an integrated system of electric and magnetic fields was difficult for previous soft robots to achieve. This is a significant improvement.

The most common propulsion method for MNRs is to use external magnetic fields and to embed permanent magnets or magnetic materials in the MNRs [28,68–70]. However, due to lack of electrical energy, these methods did not allow complex tasks to be performed, such as the collection of information. Kim et al. [71] proposed a microrobot that could use a WPT system to generate propulsion force and receive electrical energy [16,71–73]. Compared with previous research models, both power transfer efficiency and power generation efficiency have been improved. In a time-variant magnetic field, WPT was used to apply force on magnetic materials to propel and rotate microrobots. The force was proportional to the magnetic field gradient and had nothing to do with the induced current, so the resonance frequency could be used. The magnetic material designed by Kim’s team did not rely on the Lorentz force, avoiding the influence of the induced current on the force of the magnetic material. Therefore, it was more convenient to use the resonant frequency [42]. It yielded the maximum power transmission efficiency and promoted the development of microrobots of a smaller size. Also, due to the addition of magnetic materials, the magnetic coupling strength increases. Because WPT is essentially the transmission of electric energy caused by magnetic coupling between coils, a magnetic material with high relative permeability yielded a larger coupling coefficient and, thus, a higher power transfer. In addition, by adjusting the angle of the incident magnetic field and the magnetic material, the microrobot could be rotated. This kind of microrobot with WPT has many excellent characteristics. For example, it is easy to miniaturize; it can be powered by the WPT system to perform complex tasks, and it can be applied to WPT systems with any operating frequency range.

Targeted treatment and controlled drug delivery with MNRs have been achieved [74–75]. For locomotion and drug delivery, the same external power sources should be chosen, if possible. Chen et al. [76] proposed a hybrid magnetoelectric nanowire for MNR applications, which could use magnetism to assist targeted drug delivery in vitro. They designed and manufactured wire-shaped magnetoelectric MNRs that could perform wireless locomotion and on-site triggered release of therapeutics under the action of a single external power source (i.e., a magnetic field). The designed hybrid magnetoelectric core–shell composite nanowires had a magnetostrictive core and a piezoelectric shell, and it exhibited a strain-mediated magnetoelectric effect. In terms of device design and manufacturing, this biphasic core–shell configuration offered greater flexibility than single-phase magnetoelectric materials. In the presence of an external magnetic field, a piezoresponse force microscope (PFM) could be used to directly probe the ferroelectricity and magnetoelectricity of this nanowire. Experiments were carried out in a specially designed setup with three pairs of orthogonal electromagnetic coils to trigger drug release. The fabrication method of Chen’s team yielded not only easily adjustable length and diameter of the nanowires, but also excellent interface coupling between the piezoelectric and magnetostrictive phases. It enabled precise magnetic manipulation on patterned surfaces and 3D swimming in low-amplitude rotating magnetic fields. This design represents a further development of miniaturized magnetoelectric platforms for use in the biomedical field.

Following Chen et al. [76], Yang et al. [77] developed an automated microrobot platform that could quickly detect toxins. Their team developed an electromagnetic coil system (MagDisk) integrated into a fluorescence microscope and a fluorescent magnetic spore-based microrobot (FMSM). The MagDisk was low-cost and plug-and-play, and consisted of five independent coils. The maximum magnetic field strength of the MagDisk was around 20 mT. The FMSM had a two-layer nanoparticle coating. In order to drive the FMSM by an external magnetic field, Yang et al. coated magnetic Fe_3_O_4_ nanoparticles onto the outside surface of the spores for magnetization, and conjugated carbon dots onto the outside and inside walls of the magnetic spores. The MagDisk enabled actuation, control, and observation of the FMSM. In most mobile sensing applications, microrobots are driven by chemical fuels such as hydrogen peroxide (H_2_O_2_) and surfactants. In contrast, magnetic drives have good biocompatibility and external power supply. For example, a porous microelectrode constructed of MOFs proposed by Yang et al. [78] was powered by enzymes, in which MOF crystals use H_2_O_2_ as fuel to achieve bubble propulsion. A rotating magnetic field generated by the MagDisk was applied to drive the FMSM. The translational motion direction of the FMSM was determined by the yaw angle. The translation velocity was controlled by the rotation frequency and the pitch angle. The magnetic field enabled the FMSM to achieve a two-dimensional tumbling motion near the flat non-slip boundary. The magnetic gradient of the electromagnetic control system and the vertical magnetic force exerted on the FMSM were negligible. Yang’s team has provided a rapid and low-cost detection technique for medical testing in future clinical applications.

### Magnetic drive structures used in MNRs

MNRs could move in the human body with little restrictions to reach confined and delicate parts. This offers the potential of precise and minimally invasive treatment. As early as ten years ago, researchers have constructed a helical micro/nanopropeller, which was driven by an external magnetic field [79]. Ceylan et al. [42] designed a double helix architecture that could swim in a rotating magnetic field and transport cargo. The structure provided the magnetic torque of motion through biologically functionalized superparamagnetic iron oxide nanoparticles and environmentally responsive microswimmers of 3D-printed nanocomposite magnetic precursors. Due to the characteristics of the double helix, the rotational motion and translational motion of the microswimmers were coupled. The double helix structure here required a rotating magnetic field to achieve rotation. The team used a custom six-coil electromagnetic device to create the required magnetic field. They used this rotating magnetic field to apply a computer-controlled magnetic torque on the long axis of the microrobot, which generated torque on the microswimmer through a magnetic axis defined perpendicular to the helical axis. The double helix could compensate instabilities of the trajectory, which can improve the hydrodynamic efficiency to meet the power demand. It is a feasible design solution.

**Figure 2:**
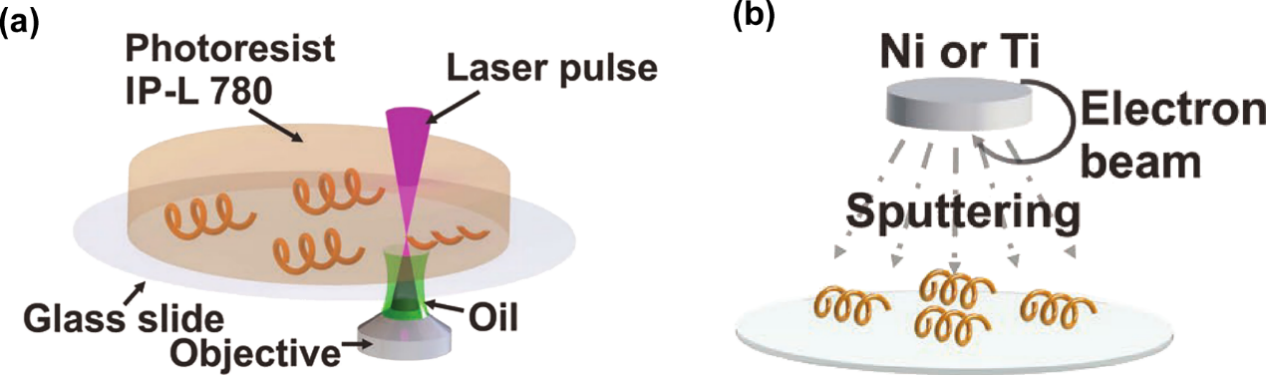
(a) A spiral swimmer with 2PP (also called artificial bacterial flagella (ABF)) and (b) coating the swimmers with nickel and titanium to make it magnetic and biocompatible. (a) and (b) adapted from [83], X. Wang et al., “MOFBOTS: Metal-Organic‐Framework‐Based Biomedical Microrobots”, Adv. Mater., with permission from John Wiley and Sons. Copyright © 2019 WILEY-VCH Verlag GmbH & Co. KGaA, Weinheim. This content is not subject to CC BY 4.0.

As the size of swimming robot enters the microscale/nanoscale, its viscous force began to dominate over the inertial force [42]. In order to deal with such problems, microorganisms in nature, such as bacteria, have evolved elaborate locomotion strategies, which use flagella to perform helical rotation. So far, people have been inspired to design many synthetic swimmers [80–81]. The use of an external magnetic field could guide magnetically driven MOFs. This locomotion mechanism was mainly limited to magnetic dragging and required a high magnetic field gradient [82]. Wang et al. [83] successfully fabricated a biocompatible and pH-responsive magnetic helical microstructure coated with zinc-based MOF and zeolite imidazole framework-8 (ZIF-8). They also developed a helical swimmer with 2PP, also called artificial bacterial flagella, as shown in Figure 2. In order to make this MNR magnetic and biocompatible, they applied nickel and titanium as coating. Because of its special microhelix structure, the robot could be flexibly controlled already by a weak external magnetic field. Under certain environmental conditions (e.g., in a complex microfluidic channel network in cell culture medium), if the trajectory of the target motion is preset, it could follow the path and transfer the payload with the help of the rotating magnetic field. Different from the device in [42], an electromagnetic control system composed of three pairs of Helmholtz coils was used to generate an alternative rotational magnetic field [84]. The frequency of the rotating magnetic field affected the propulsion speed of the helical swimming robot. To a certain extent, the higher the frequency, the higher the speed.

**Figure 3:**
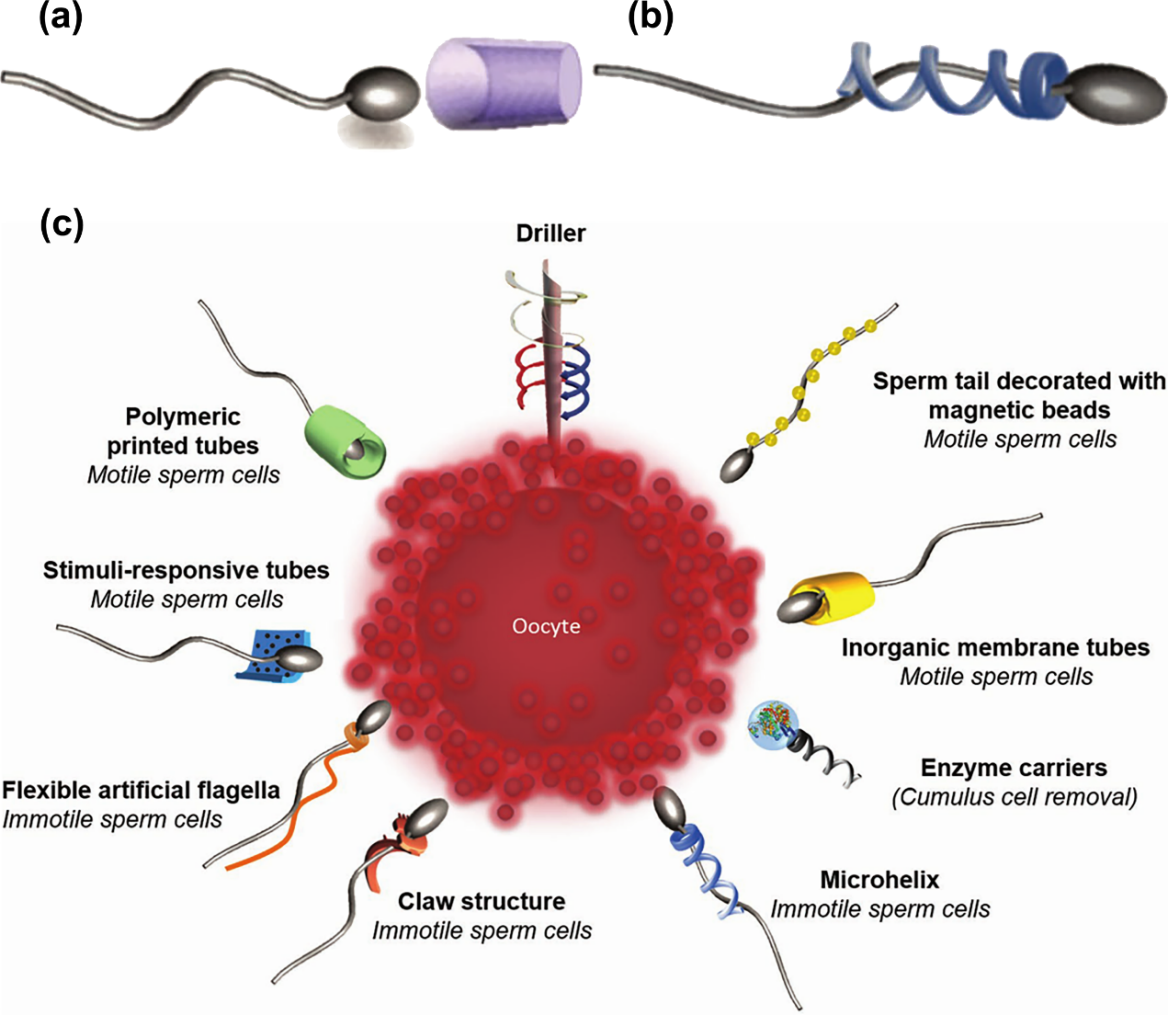
(a) Tubular, (b) helical, and (c) novel microswimmer designs for fertilization. Clockwise from top: magnetically actuated drillers for penetrating the cumulus layer of the oocyte; sperm tail decorated with magnetic beads for magnetic actuation of the tail; rolled-up microtubes made of inorganic nanomembranes for the guidance of motile sperm cells; magnetic helical enzyme carriers for the cumulus cell removal by enzymes (e.g., hyaluronidase); artificial flagella with multiple windings and a claw-shaped sperm carrier for the delivery of immotile sperm cells; artificial flexible flagella for transport of immotile spermatozoa; stimuli-responsive polymeric microtubes for the delivery of motile spermatozoa; and polymeric printed microtubes for the guidance of motile sperm cells. Panels (a–c) adapted from [85], V. Magdanz et al., “Spermatozoa as Functional Components of Robotic Microswimmers”, Adv. Mater., with permission from John Wiley and Sons. Copyright © 2017 WILEY-VCH Verlag GmbH & Co. KGaA, Weinheim. This content is not subject to CC BY 4.0.

Magdanz et al. [85] reported two MNRs similar to artificial bacterial flagella, namely tubular and helical spermbots, as shown in Figure 3. In addition, other forms of design for new miniature swimmers targeting fertilization were presented. The helical spermbots were propelled by a rotating magnetic field and could capture, transport, and deliver immotile sperm cells [86]. Studies have been shown that prolonged exposure to high magnetic field strengths will reduce sperm motility, but relatively short periods of exposure to low magnetic fields have little effect on cells. Low-strength external magnetic fields have been used to control tubular and helical spermbots [86]. The tubular spermbots reported here were guided by external magnets and sperm cells, and used sperm cells to push microtubules. The flagellum of this flexible artificial MNRs was basically a magnetic microhelix that was actuated via an external rotating magnetic field. These magnetic microspirals could move in the fluid under the influence of the rotating magnetic field. Higher viscosity fluids made the helix-like shaped MNRs swim faster. In order to further promote the functionalization of biomolecules, a layer of titanium was deposited on the outside, which not only served as a protective layer, but also yielded biocompatibility. At the same time, adding a very thin layer of nickel or iron could improve magnetic properties. In order not to affect the swimming speed and let the flagella oscillate naturally as much as possible, Magdanz and others believed that it is possible to make strongly magnetized conical tubes or ring-like microstructures. The structure with sharp tip could also penetrate the cumulus layer surrounding the oocyte. At the same time, it was also necessary to apply a higher magnetic field gradient to guide the magnetic structure with superparamagnetic beads. Artificial magnetic helices have been shown to be used as drug carriers delivering liposomes loaded with drugs or DNA to single cells [80]. This reflects the prominent role of tubular and helical spermbots and microrobotic for research on medical treatment.

Different from the magnetic tubular and helical structures, Kim and others studied a microrobot that could use a WPT system to generate propulsion force and receive electrical energy with a bar-type magnetic material [71] that could achieve a higher magnetic field gradient. In comparison, under the same magnitude of the magnetic field and with the same material properties, the bar shape along the transmitting coil proposed by the team could generate greater torque, and the proposed model could be controlled or rotated by the direction of the external magnetic field. In addition, compared to the square-shaped PG microrobot, the star-shaped PG microrobot also showed greater stability and faster rate of stabilization [87]. There are also differences in the form of the magnetic field. Chen et al. [76] introduced a type of conical rotating magnetic field for actuating a 3D propulsion mode for nanowires, when studying hybrid magnetoelectric nanowires for nanorobotic applications. The magnetic nanowires were synchronized with the conical field, rotating around the axis of rotation on the conical surface. Because the upper and lower parts of the conical surface were asymmetric, this caused translational forces on the nanowires, which was a way to drive rigid nanowires. In order to change the swimming direction, the direction of the rotation axis could be controlled by adjusting the pitch angle, and different control effects could be achieved. Table 3 shows several different shapes of magnetic drive structures.

**Table 3:** Magnetic drive structures of different shapes.

Shape	Magnetic field	Effect	Specific structure	Advantage	Ref.

helical structure	custom six-coil electromagnetic setup to create rotating magnetic fields for driving	volumetric cargo loading and swimming capabilities	double helix architectures; biofunctionalized superparamagnetic iron oxide nanoparticles provide magnetic torque for movement	can compensate trajectory instabilities, which is in favor of the hydrodynamic efficiency and thus power requirements	[42]
	electromagnetic control system composed of three pairs of Helmholtz coils generating an alternative rotational magnetic field	single-cell targeting can be achieved in cell culture media and the controlled delivery of cargo can be achieved inside a complex microfluidic channel network	magnetic helical microstructure coated with a zinc-based MOF, ZIF-8, all coated with nickel and then titanium	biocompatibility and pH-responsivity; by increasing the frequency of the rotating magnetic field, the propulsion speed of the helical swimmer could be increased	[83]
tubular structure	tubular spermbots guided by an external magnet and microtubes propelled by sperm cells	guidance and transportation of sperm cells	photolithographic fabrication, coated with a very thin layer of nickel or iron to improve magnetic properties and titanium as a protective layer	biocompatibility, biomolecule functionalization	[85]
bar-type structure	control and rotation through the direction of the external magnetic-field	higher magnetic-field gradient	magnetic materials (ferrite sheet)	excellent in generating torque	[71]
cone structure	guided by an external magnet	capture, transport, and release sperm and other cells; magnetic driller to help penetrate the outer layer of cells	improvement idea on the tubular structure	does not interfere with the natural flagella beating and is relatively tubular to avoid velocity reduction	[85]

## Conclusion

This review summarizes the latest research on magnetic MNRs and discusses the development of MNRs regarding magnetic materials, magnetoelectric concepts, and magnetic drive structures. Magnetic fields have been used in the research of MNRs in various forms. Magnetic MNRs may become the research direction of a new generation of MNRs due to their excellent characteristics in motion control, targeted transportation of goods, and energy transmission. With the continuous development of magnetic micro- and nanorobotic technology, we can observe the continuous improvement and perfection of the MNPs and the miniaturization of magnetoelectrical devices and magnetic drive structures integrated into the robots. This greatly improves the efficiency of power transmission of the MNR, the sensitivity of the motion control, and the biocompatibility, with better performance and wider applications. In-depth research in the field of MNRs is inseparable from that on magnetic fields. Although magnetic fields are used to enhance the technology of MNRs and solve problems that were difficult or unresolved by other methods before, the application of magnetic fields still needs continuous exploration and improvement to expand the application of magnetic MNRs in biomedicine, electronic technology, and other fields. In the near future, the technology of magnetic MNRs will be more advanced and the scope of application will be further expanded.

## References

[1] Li M, Xi N, Wang Y, Liu L (2021). IEEE Trans Biomed Eng.

[2] Medina-Sánchez M, Magdanz V, Guix M, Fomin V M, Schmidt O G (2018). Adv Funct Mater.

[3] Vikram Singh A, Sitti M (2016). Curr Pharm Des.

[4] Yang Z, Gu C, Chen T, Hu C, Sun L (2018). IEEE Trans Nanotechnol.

[5] Ricotti L, Menciassi A (2015). J Nanopart Res.

[6] Villa K, Pumera M (2019). Chem Soc Rev.

[7] Wang J, Xiong Z, Zheng J, Zhan X, Tang J (2018). Acc Chem Res.

[8] Wu C, Feng J, Peng L, Ni Y, Liang H, He L, Xie Y (2011). J Mater Chem.

[9] Hu S, Hu R, Dong X, Wei T, Chen S, Sun D (2019). Opt Express.

[10] Feng L, Di P, Arai F (2016). Int J Rob Res.

[11] Lu X, Zhao K, Liu W, Yang D, Shen H, Peng H, Guo X, Li J, Wang J (2019). ACS Nano.

[12] Xu L, Gong D, Chen K, Cai J, Zhang W (2020). J Appl Phys.

[13] Fan X, Sun M, Lin Z, Song J, He Q, Sun L, Xie H (2018). IEEE Trans Nanotechnol.

[14] Li J, Wang H, Shi Q, Zheng Z, Cui J, Sun T, Ferraro P, Huang Q, Fukuda T (2020). IEEE Trans Nanotechnol.

[15] Yang L, Wang Q, Zhang L (2018). IEEE Trans Nanotechnol.

[16] Kim D, Hwang K, Park J, Park H H, Ahn S (2017). IEEE Trans Magn.

[17] Wei T, Liu J, Li D, Chen S, Zhang Y, Li J, Fan L, Guan Z, Lo C-M, Wang L (2020). Small.

[18] Sitti M, Wiersma D S (2020). Adv Mater (Weinheim, Ger).

[19] Feng L, Liang S, Zhou X, Yang J, Jiang Y, Zhang D, Arai F (2017). Appl Phys Lett.

[20] Park J, Jin C, Lee S, Kim J-Y, Choi H (2019). Adv Healthcare Mater.

[21] Xu T, Gao W, Xu L-P, Zhang X, Wang S (2017). Adv Mater (Weinheim, Ger).

[22] Chen J, Wang Y (2020). Nanotechnology.

[23] Mellal L, Belharet K, Folio D, Ferreira A (2015). J Nanopart Res.

[24] Alcântara C C J, Kim S, Lee S, Jang B, Thakolkaran P, Kim J-Y, Choi H, Nelson B J, Pané S (2019). Small.

[25] Li H, Zhang J, Zhang N, Kershaw J, Wang L (2016). J Microencapsulation.

[26] Martel S (2015). J Nanopart Res.

[27] Bakshi S F, Guz N, Zakharchenko A, Deng H, Tumanov A V, Woodworth C D, Minko S, Kolpashchikov D M, Katz E (2018). Nanoscale.

[28] Jang G B, Jeon S, Nam J, Lee W, Jang G (2015). IEEE Trans Magn.

[29] Chatzipirpiridis G, Ergeneman O, Pokki J, Ullrich F, Fusco S, Ortega J A, Sivaraman K M, Nelson B J, Pané S (2015). Adv Healthcare Mater.

[30] Martel S (2016). Biomicrofluidics.

[31] Jahnavi V S, Tripathy S K, Rao A V N R (2020). J Electron Mater.

[32] Zhang L, Li Y, Yu J C, Chan K M (2016). RSC Adv.

[33] Cheang U K, Kim M J (2015). J Nanopart Res.

[34] Cho J-H, Kim M-S (2015). J Magn.

[35] Yu J, Yang L, Zhang L (2018). Int J Rob Res.

[36] Zakharchenko A, Guz N, Laradji A M, Katz E, Minko S (2018). Nat Catal.

[37] Klumpp S, Kiani B, Vach P, Faivre D (2015). Phys Scr.

[38] Doherty C M, Knystautas E, Buso D, Villanova L, Konstas K, Hill A J, Takahashi M, Falcaro P (2012). J Mater Chem.

[39] Nayak S, Horcajada Cortés P, Rojas Macías S (2021). Water purification: Removal of Heavy metals Using Metal-Organic Frameworks (MOFs). Metal-Organic Frameworks in Biomedical and Environmental Field.

[40] Yu J, Xu T, Lu Z, Vong C I, Zhang L (2017). IEEE Trans Rob.

[41] Peters C, Hoop M, Pané S, Nelson B J, Hierold C (2016). Adv Mater (Weinheim, Ger).

[42] Ceylan H, Yasa I C, Yasa O, Tabak A F, Giltinan J, Sitti M (2019). ACS Nano.

[43] Agostinelli E, Vianello F, Magliulo G, Thomas T, Thomas T J (2015). Int J Oncol.

[44] Li T, Li J, Morozov K I, Wu Z, Xu T, Rozen I, Leshansky A M, Li L, Wang J (2017). Nano Lett.

[45] Mahmoudi M, Hofmann H, Rothen-Rutishauser B, Petri-Fink A (2012). Chem Rev.

[46] Kučírková L, Královec K, Havelek R, Bruckova L, Sedlak M (2015). Chem Listy.

[47] Uvet H, Demircali A A, Kahraman Y, Varol R, Kose T, Erkan K (2018). Micromachines.

[48] Gopal N O, Lo H-H, Ke S-C (2008). J Am Chem Soc.

[49] Martel S, Mohammadi M, Felfoul O, Lu Z, Pouponneau P (2009). Int J Rob Res.

[50] Folio D, Ferreira A (2017). IEEE Trans Rob.

[51] Arcese L, Fruchard M, Ferreira A (2013). IEEE Trans Rob.

[52] Shanker J, Buchi Suresh M, Narsinga Rao G, Suresh Babu D (2019). J Mater Sci.

[53] Chen M, Mishra R, Wu Y, Lee K, Yang H (2018). Adv Opt Mater.

[54] Falcaro P, Lapierre F, Marmiroli B, Styles M, Zhu Y, Takahashi M, Hill A J, Doherty C M (2013). J Mater Chem C.

[55] Zhou H, Mayorga-Martinez C C, Pané S, Zhang L, Pumera M (2021). Chem Rev.

[56] Yang Z, Zhang L (2020). Adv Intell Syst.

[57] Chen X-Z, Hoop M, Mushtaq F, Siringil E, Hu C, Nelson B J, Pané S (2017). Appl Mater Today.

[58] Ceylan H, Giltinan J, Kozielski K, Sitti M (2017). Lab Chip.

[59] Qiu F, Nelson B (2015). Engineering.

[60] Patiño T, Arqué X, Mestre R, Palacios L, Sánchez S (2018). Acc Chem Res.

[61] Fu Q, Guo S, Yamauchi Y, Hirata H, Ishihara H (2015). Biomed Microdevices.

[62] Jeong S, Choi H, Choi J, Yu C, Park J-O, Park S (2010). Sens Actuators, A.

[63] Jeong S, Choi H, Go G, Lee C, Lim K S, Sim D S, Jeong M H, Ko S Y, Park J-O, Park S (2016). Med Eng Phys.

[64] Kim D-i, Song S, Jang S, Kim G, Lee J, Lee Y, Park S (2020). Smart Mater Struct.

[65] Zolfagharian A, Kouzani A Z, Khoo S Y, Nasri-Nasrabadi B, Kaynak A (2017). Sens Actuators, A.

[66] Li Y, Sun Y, Xiao Y, Gao G, Liu S, Zhang J, Fu J (2016). ACS Appl Mater Interfaces.

[67] Migliorini L, Santaniello T, Yan Y, Lenardi C, Milani P (2016). Sens Actuators, B.

[68] Hunter E E, Brink E W, Steager E B, Kumar V (2018). IEEE Rob Autom Lett.

[69] Go G, Choi H, Jeong S, Lee C, Ko S Y, Park J O, Park S (2015). IEEE Trans Magn.

[70] Yang L, Wang Q, Vong C-I, Zhang L (2017). IEEE Rob Autom Lett.

[71] Kim D, Park J, Park B, Shin Y, Kim K, Park H H, Ahn S (2020). IEEE Trans Magn.

[72] Kim D, Park J, Park H H, Ahn S (2015). IEEE Trans Magn.

[73] Kim D, Kim M, Yoo J, Park H-H, Ahn S (2015). J Appl Phys.

[74] Fusco S, Huang H-W, Peyer K E, Peters C, Häberli M, Ulbers A, Spyrogianni A, Pellicer E, Sort J, Pratsinis S E (2015). ACS Appl Mater Interfaces.

[75] Lee H, Choi H, Lee M, Park S (2018). Biomed Microdevices.

[76] Chen X-Z, Hoop M, Shamsudhin N, Huang T, Özkale B, Li Q, Siringil E, Mushtaq F, Di Tizio L, Nelson B J (2017). Adv Mater (Weinheim, Ger).

[77] Yang L, Zhang Y, Wang Q, Zhang L (2020). IEEE Trans Biomed Eng.

[78] Yang Y, Arqué X, Patiño T, Guillerm V, Blersch P-R, Pérez-Carvajal J, Imaz I, Maspoch D, Sánchez S (2020). J Am Chem Soc.

[79] Ma X, Sánchez S (2017). Nanomedicine (London, U K).

[80] Qiu F, Fujita S, Mhanna R, Zhang L, Simona B R, Nelson B J (2015). Adv Funct Mater.

[81] Lum G Z, Ye Z, Dong X, Marvi H, Erin O, Hu W, Sitti M (2016). Proc Natl Acad Sci U S A.

[82] Elsaidi S K, Sinnwell M A, Banerjee D, Devaraj A, Kukkadapu R K, Droubay T C, Nie Z, Kovarik L, Vijayakumar M, Manandhar S (2017). Nano Lett.

[83] Wang X, Chen X-Z, Alcântara C C J, Sevim S, Hoop M, Terzopoulou A, de Marco C, Hu C, de Mello A J, Falcaro P (2019). Adv Mater (Weinheim, Ger).

[84] Yu C-H, Kim S H (2016). J Magn.

[85] Magdanz V, Medina-Sánchez M, Schwarz L, Xu H, Elgeti J, Schmidt O G (2017). Adv Mater (Weinheim, Ger).

[86] Medina-Sánchez M, Schwarz L, Meyer A K, Hebenstreit F, Schmidt O G (2016). Nano Lett.

[87] Feng L, Zhang S, Jiang Y, Zhang D, Arai F (2017). J Appl Phys.

